# Rapid antimicrobial susceptibility test for identification of new therapeutics and drug combinations against multidrug-resistant bacteria

**DOI:** 10.1038/emi.2016.123

**Published:** 2016-11-09

**Authors:** Wei Sun, Rebecca A Weingarten, Miao Xu, Noel Southall, Sheng Dai, Paul Shinn, Philip E Sanderson, Peter R Williamson, Karen M Frank, Wei Zheng

**Affiliations:** 1 National Center for Advancing Translational Sciences, National Institutes of Health, Bethesda, MD 20892, USA; 2 Department of Laboratory Medicine, National Institutes of Health Clinical Center, Bethesda, MD 20892, USA; 3 Laboratory of Clinical Infectious Diseases, National Institute of Allergy and Infectious Diseases, National Institutes of Health, Bethesda, MD 20892, USA

**Keywords:** antimicrobial susceptibility testing, drug repositioning, drug repurposing, *Klebsiella pneumoniae*, multidrug-resistant bacteria, targeted drug combination, ultra-high-throughput screen

## Abstract

Current antimicrobial susceptibility testing has limited screening capability for identifying empirical antibiotic combinations to treat severe bacterial infections with multidrug-resistant (MDR) organisms. We developed a new antimicrobial susceptibility assay using automated ultra-high-throughput screen technology in combination with a simple bacterial growth assay. A rapid screening of 5170 approved drugs and other compounds identified 25 compounds with activities against MDR *Klebsiella pneumoniae*. To further improve the efficacy and reduce the effective drug concentrations, we applied a targeted drug combination approach that integrates drugs' clinical antimicrobial susceptibility breakpoints, achievable plasma concentrations, clinical toxicities and mechanisms of action to identify optimal drug combinations. Three sets of three-drug combinations were identified with broad-spectrum activities against 10 MDR clinical isolates including *K. pneumoniae*, *Acinetobacter baumannii*, *Pseudomonas aeruginosa*, *Citrobacter freundii*, *Enterobacter cloacae* and *Escherichia coli*. Colistin–auranofin–ceftazidime and colistin–auranofin–rifabutin suppressed >80% growth of all 10 MDR strains; while rifabutin–colistin–imipenem inhibited >75% of these strains except two *Acinetobacter baumannii* isolates. The results demonstrate this new assay has potential as a real-time method to identify new drugs and effective drug combinations to combat severe clinical infections with MDR organisms.

## Introduction

Infections with multidrug-resistant (MDR) organisms have emerged as a significant worldwide public health crisis, with two million infections and an estimated 23 000 deaths in the United States annually.^1^ The incidence is increasing partially due to the selective pressure from widespread use of antibiotics in both humans and animals.^2^ Current treatment of bacterial infections commonly requires broad-spectrum antibiotics until a pathogen can be isolated, identified and antimicrobial susceptibility testing performed, which can take a few days.^3^ The commonly used susceptibility testing for clinical diagnostics include broth microdilution, agar dilution, rapid automated instrument methods, disk diffusion and gradient diffusion methods. These methods are usually suitable for testing up to ~25 antibiotics for a given sample. There is limited capability for testing drug combinations, although combination therapy is used routinely to treat severe infections.^4, 5^ Therefore, an improvement of the current antimicrobial susceptibility testing methods is needed to meet the challenge of treatment of infections caused by MDR bacteria (MDRB).^6^ New susceptibility testing methodologies are currently being explored to address this concern.^7^ Rapid identification of effective antibiotics is critical in clinical practice because the mortality rate of patients can increase as high as 9% for every hour the effective antimicrobial treatment is delayed.^8, 9^

It takes an average of 10–12 years and abundant resources to develop a new antibiotic.^10, 11^ The use of drug repurposing screens against individual patient isolates, on the other hand, is an alternative approach to identify effective therapeutics against infections with MDRB. For example, the repurposing of existing drugs has identified promising antibiotics against MDR *Acinetobacter baumannii* and *Borrelia burgdorferi*.^12, 13, 14^ Reports has described <200 approved antibiotics that are available to clinicians to select for treatments.^11, 15^ In addition, there are thousands of additional approved drugs for diseases other than infection that may have activities against MDRB^16^ or may potentially re-sensitize the MDRB to standard care antibiotics by overcoming a specific drug-resistant mechanism. However, it is challenging to use the standard clinical antimicrobial susceptibility methods for screening of hundreds of these antibiotics and other approved drugs to identify effective therapeutics against MDRB.

To address the limitations of current methods, we have developed an automated ultra-high-throughput bacterial growth assay (HIGA) that combines a bacterial growth assay with automated quantitative high-throughput screening technology in a miniaturized 1536-well plate format.^17, 18, 19, 20^ HIGA is robust and low cost, as it measures the light absorbance (OD_600_) of bacterial growth without the necessity of other detection reagents. This new method enables the testing of hundreds of antibiotics in a concentration–response manner as well as in complicated drug combinations. The compound susceptibility results can be obtained within 2 days of receiving clinical isolates. Because it is based on a phenotypic screen, HIGA can provide preliminary results for unidentified clinical isolates without knowing the unidentified mechanism of resistance or other susceptibility results. Therefore, HIGA has the potential to be used broadly in a clinical setting for antimicrobial susceptibility testing to identify effective individual therapeutics and/or effective drug combinations against MDRB.^21^

## Materials and Methods

### Materials

All chemicals and antibiotics were purchased from Sigma-Aldrich (St Louis, MO, USA) and US Pharmacopeial Convention (Rockville, MD, USA). The ATP content kit (BacTiter-Glo, catalog number G8230) was purchased from Promega (Madison, WI, USA).

### Preparation of drug-resistant bacterial strains for high-throughput screen

Bacterial strains are listed in Table 1 and were isolated from patients at the Clinical Center of National Institutes of Health (NIH) or obtained from the College of American Pathologists Breakpoint Toolkit. Bacteria were routinely cultured on blood agar plates (Remel, Lenexa, KS, USA), and individual colonies were inoculated into freshly prepared tryptic soy broth (TSB; Thermo Fisher Scientific, Waltham, MA, USA) and grown with aeration at 37 °C. Once cultures reached an absorbance (600 nm) of 0.25–0.3, 900 μL of culture was added to 100 μL of sterile glycerol, and was vortexed, aliquoted and stored at −80 °C.

### Antibacterial absorbance assay and ATP content assay

Frozen isolates were thawed at 4 °C and serially diluted in pre-made TSB from 1/100 to 1/1000 in a 1536-well plate. The cultures were treated with drugs and incubated at 37 °C, 5% CO_2_ humidified atmosphere for 2h–24 h, when indicated. Absorbance of cultures was monitored at OD_600_ to determine the drug susceptibility. BacTiter-Glo assay, the ATP content assay to measure bacterial viability, was performed as per manufacturer instructions and was conducted similarly as the absorbance assay. In the detection step, 4 μL BacTiter-Glo detection reagent was added to each well and the plates were incubated at room temperature for 15 min before the detection of luminescence intensity in a ViewLux plate reader (PerkinElmer, Waltham, MA, USA).

### Compound library and liquid handling instrument

The library of 1280 pharmacologically active compounds (LOPAC1280) was purchased from Sigma-Aldrich. The NIH Chemical Genomics Center Pharmaceutical (NPC) collection was collected in-house through compounds purchased and custom synthesis.^22^ Briefly, the NPC library consists of drugs approved for human or animal use by the US Food and Drug Administration (FDA; 49%), drugs approved in Canada/UK/EU/Japan (23%) and compounds used in clinical trials or compounds commonly used in biomedical research (28%). Compounds from all libraries were obtained as powder samples and dissolved in dimethylsulphoxide, except a few water soluble antibiotics were freshly dissolved in diH_2_O before screening. It is important to determine the purity of compounds (quality control) before compound screening.

Compound screening experiments were performed as previously described.^23^ Briefly, 2.5 μL TSB was dispensed into each well of 1536-well clear bottom black plates using the Multidrop Combi (Thermo Fisher Scientific) followed by 23 nL compound transferred by the NX-TR Pintool (WAKO Scientific Solutions, San Diego, CA, USA). A volume of 2.5 μL per well of bacterial culture was dispensed with a final dilution 1:500 using the Multidrop Combi (Thermo Fisher Scientific). After incubation for 16 h at 37 °C, 5% CO_2_, the plates were read in an absorbance detection mode (OD_600_) on a ViewLux plate reader (PerkinElmer).

### Clinically targeted synergistic drug combination assay

In each combination set, two or three drugs were added into a single well for bacterial viability tests. In two-drug combination experiments, drug #1 was near or below its C_max_ (the maximal concentration of drug in human plasma), and combined with a titration series of 11 concentrations of drug #2 to generate a targeted drug combination (TDC) dose–response curve for drug number 2. The concentration–response curves of bacterial viability results (OD_600_) for drug number 2 were generated in the presence or absence of drug number 1. Any greater than fivefold left-shift of the IC_50_ value for drug number 2 was considered a preliminarily significant effect that was further analyzed. In three-drug combination assays, drug number 1 and drug number 2 were set at or below their *C*_max_ values, and concentration response curves of drug number 3 were generated in the presence or absence of drug number 1 and drug number 2. The results were analyzed similarly as the two-drug combinations.

### Determination of minimum inhibitory concentration and fractional inhibitory concentration index

The minimum inhibitory concentrations (MICs) of individual antibiotics were determined by standard broth microdilution assay according to the Clinical and Laboratory Standards Institute guidelines.^24^ The fractional inhibitory concentration (FIC) index was determined by testing a range of drug combinations between 1/128 the MIC to two times the MIC. The FIC index was calculated as [(MIC of drug A in combination/MIC of drug A alone)]+[(MIC of drug B in combination/MIC of drug B alone)] and so on. Synergy was defined as FIC index ≤0.5; indifference as FIC index between >0.5 to <4; and antagonism if the FIC index ≥4, *n*≥3.

### Data analysis

The primary screen data were analyzed using customized software developed internally at National Center for Advancing Translations Sciences (NCATS). IC_20_, IC_50_ and IC_90_ values were calculated using the Prism software (Graphpad Software, Inc. San Diego, CA, USA). Bliss independence with Prism was used to define synergistic or additive effects. The data were fitted sharing all the parameters, using ‘log (inhibitor) vs response–variable slope (four parameters)'. Criteria of IC_50_ change greater than fivefold was used to define potentially synergistic effects for the evaluation.^25^

## Results

### HIGA assay for antimicrobial susceptibility testing in 1536-well plate format

**Figure 1:**
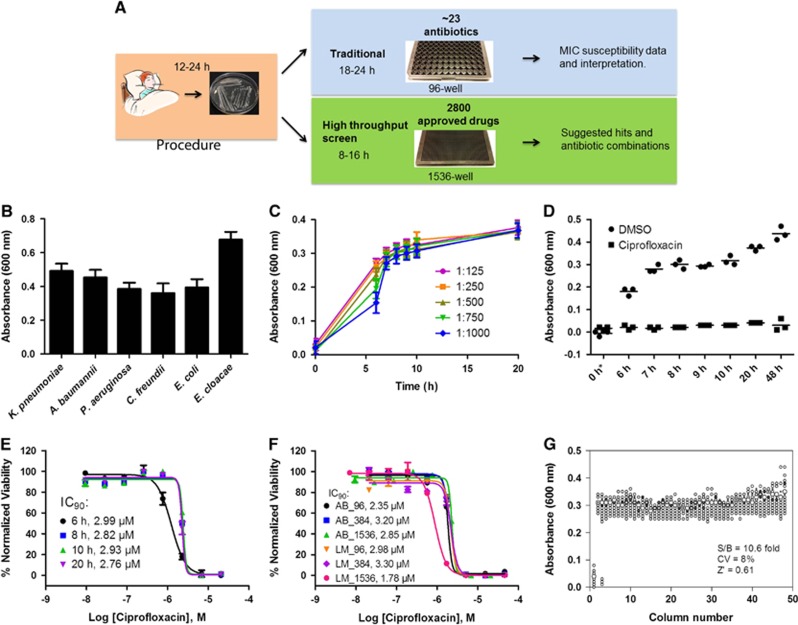
Development and validation of automated high-throughput bacterial growth assay (HIGA). (**A**) Diagram of standard susceptibility assay and high-throughput antibiotic screening method timelines. (**B**) Endpoint growth assay for *K*. *pneumoniae* (KPNIH301), *A. baumannii* (ABNIH144), *P. aeruginosa* (PANIH338), *C. freundii* (CFB10), *E. cloacae* (ECB2) and *E. coli* (ECOB11); *n*=32. Bars represent mean, and error bars represent the SD. (**C**) Growth curve of *K. pneumoniae* KPNIH301 in 1536-well plate. Frozen KPNIH301 were diluted to different starting ratios and incubated at 37 °C. Data points represent the mean, and error bars represent the SD; *n*=3. (**D**) Time course of KPNIH301 growth in the presence of 46 μM ciprofloxacin or 0.46% dimethylsulphoxide (DMSO). Data points represent individual experiments; *n*=3. *X axis is not linear. (**E**) Dose–response curves for ciprofloxacin. KPNIH301 was incubated with varying concentrations of ciprofloxacin at 37 °C for 6, 8, 10 and 20 h. The data points represent the mean, and error bars represent the SD; *n*=3. (**F**) Concentration–response curves for ciprofloxacin in 96-well, 384-well, 1536-well absorbance assay (AB) and ATP content luminescence assay (LM). KPNIH301 was incubated with different concentrations of ciprofloxacin for 8 h at 37 °C before detection at OD_600_. The data points represent the mean, and error bars represent the SD; *n*=3. (**G**) Scatter plot of the results from a DMSO plate screening. The wells in column 1 of the 1536-well assay plates contained 46 μM ciprofloxacin as a positive control (0% viability); the wells in column 3 contained varying doses of ciprofloxacin at 1:3 serial dilutions from top to bottom. The wells in the rest of plate contained DMSO as a negative control (100% viability). The signal-to-basal ratio (S/B) in this plate was 10.6-fold, with a coefficient of variation (CV) of 8% and a Z′ factor of 0.61.

To develop a broad-spectrum high-throughput method for antimicrobial susceptibility testing, we applied a previously developed quantitative high-throughput screening assay platform in a 1536-well plate.^20^ Samples of MDRB were first expanded and stored in frozen vials. The frozen stock was diluted and dispensed into 1536-well assay plates at 5 μL per well (Figure 1A) and incubated at 37 °C. The optical density at a wavelength of 600 nm (OD_600_) was measured as an indicator of bacterial growth using a CCD-imaging-based plate reader for high-throughput plate reading. Multiple absorbance readings were taken and an OD_600_ of 0.3–0.7 was normally reached after 6–24 h incubation (Figure 1B). This method was able to monitor the growth of multiple MDRB, including *Klebsiella pneumoniae*, *Acinetobacter baumannii*, *Pseudomonas aeruginosa*, *Citrobacter freundii*, *Enterobacter cloacae* and *Escherichia coli* (Figure 1B). The susceptible *K. pneumoniae* KPNIH301 strain was used for further assay optimization. KPNIH301 frozen stock was diluted in TSB at five different dilution ratios and the bacterial growth rates determined (Figure 1C). The 1/1000 dilution of bacterial stock solution was chosen as the optimal condition as KPNIH301 reached stationary growth phase in 8 h (Figure 1C). We confirmed that 46 μM ciprofloxacin, one of the antibiotics for standard therapy, inhibited growth over time (Figure 1D), and determined the concentration–response curves of ciprofloxacin (Figure 1E). Because the IC_90_ value is correlated with the MIC value of a compound, we calculated the IC_90_ value as a compound's activity against bacterial growth. The IC_90_ values (90% of maximal inhibitory concentration) of ciprofloxacin in the 1536-well plate assay ranged between 2.76 μM and 2.99 μM for 6–20 h incubation times (Figure 1E), similar to its IC_90_ values determined in 96-well plates (2.35±0.61 μM) and 384-well plates (3.20±0.11 μM) (Figure 1F). We also measured concentration–response curves of ciprofloxacin against KPNIH301 using a luminescence bacterial ATP content assay to determine viability of bacteria. IC_90_ values of ciprofloxacin in the ATP content assays were 2.98±0.04 μM in 96-well, 3.30±0.06 μM in 384-well and 1.78±0.03 μM in 1536-well plates, comparable to those determined in the absorbance assay. Taken together, the results demonstrated that HIGA is an effective method for determining compound inhibitory activity (Figure 1F and Supplementary Figure S1).^26^ To evaluate robustness and reproducibility of HIGA, the parameters for compound screening were determined. We found that the signal-to-basal ratio was 10.6-fold, CV was 8% and Z′ factor was 0.62 for the 1536-well plate assay (Figure 1G).

We also determined the inhibitory activities of a panel of antibiotics routinely used in clinical care against two resistant *K. pneumoniae* isolates (KPNIH1760 and KPNIH1776) and two susceptible *K. pneumoniae* isolates (KPNIH301 and KPNIH478) using HIGA. We found that the activities determined in our assay correlated, except in a few cases, to the MIC values measured by the automated broth microdilution method (BD Phoenix, Becton, Dickinson and Company, Franklin Lakes, NJ, USA) (Supplementary Table S1). Only imipenem for KPNIH301 and KPNIH478 had discrepancies between the HIGA screen and the microdilution method.

### Drug repurposing screen to identify effective compounds against MDR *K. pneumoniae*

**Figure 2:**
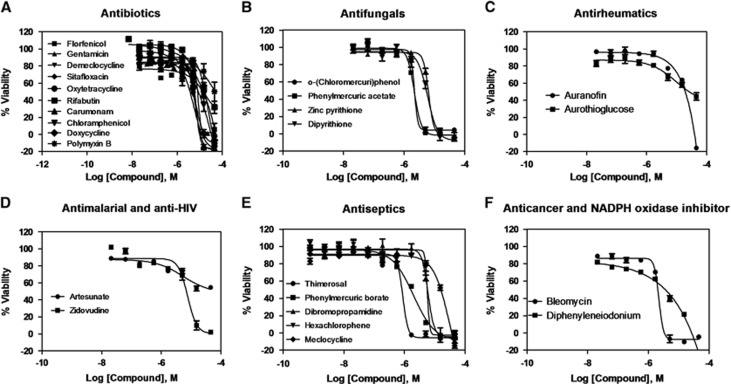
Concentration–response curves of the inhibition of *K. pneumoniae* KPNIH1760 growth by 25 identified active compounds. The 25 compounds with confirmed antibacterial activity are divided into six groups: (**A**) antibiotics; (**B**) antifungals; (**C**) antirheumatics; (**D**) antimalarial and anti-HIV; (**E**) antiseptics; (**F**) anticancer and NADPH oxidase inhibitor; *n*=4. Data points represent mean, and error bars represent the SEM.

The initial screening of two isolates (KPNIH1760 and KPNIH1776) confirmed they were resistant to 19 of 20 tested antibiotics with the exception of gentamicin. KPNIH301 and KPNIH478 were susceptible strains (Supplementary Table S1). We performed a drug repurposing screen against these four *K. pneumoniae* clinical isolates with HIGA. The compound collections tested include 3890 approved drugs^22^ and 1280 bioactive compounds^27^ in four concentrations ranging from 0.4 to 46 μM. A set of 26 compounds was identified as primary hits with criteria of IC_50_ <20 μM and maximal inhibition >50% against drug-resistant strains KPNIH1760 and KPNIH1776. The activities of 25 of 26 primary hit compounds were confirmed, demonstrating the high reproducibility of HIGA (Table 2 and Supplementary Table S2). Only one compound did not meet the criteria because its activity was higher than 20 μM in the conformational screens. These 25 compounds consist of six groups based on their known functions and clinical indications and include: ten antibiotics (gentamicin, demeclocycline, oxytetracycline, rifabutin, chloramphenicol, doxycycline, polymyxin B, florfenicol, sitafloxacin and carumonam), four antifungals (o-(chloromercuri)phenol, phenylmercuric acetate, zinc pyrithione and dipyrithione), five antiseptics (thimerosal, dibromopropamidine, phenylmercuric borate, hexachlorophene and meclocycline), two antirheumatics (auranofin and aurothioglucose), one antiviral (zidovudine), one antimalarial (artesunate), one anticancer (bleomycin) and one NADPH oxidase inhibitor (diphenyleneiodonium) (Figure 2). We further confirmed the activities of zidovudine, rifabutin, auranofin, bleomicin, polymyxin B and gentamicin against KPNIH1760 with the standard broth microdilution assay according to CLSI guidelines (Table 3A). For these six compounds, four of the MIC values were within threefold of the calculated IC_90_ data from the HIGA. Bleomycin and polymixin B showed sevenfold and fourfold differences, respectively.

### Identification of two-drug synergistic combinations against MDR *K. pneumoniae*

**Figure 3:**
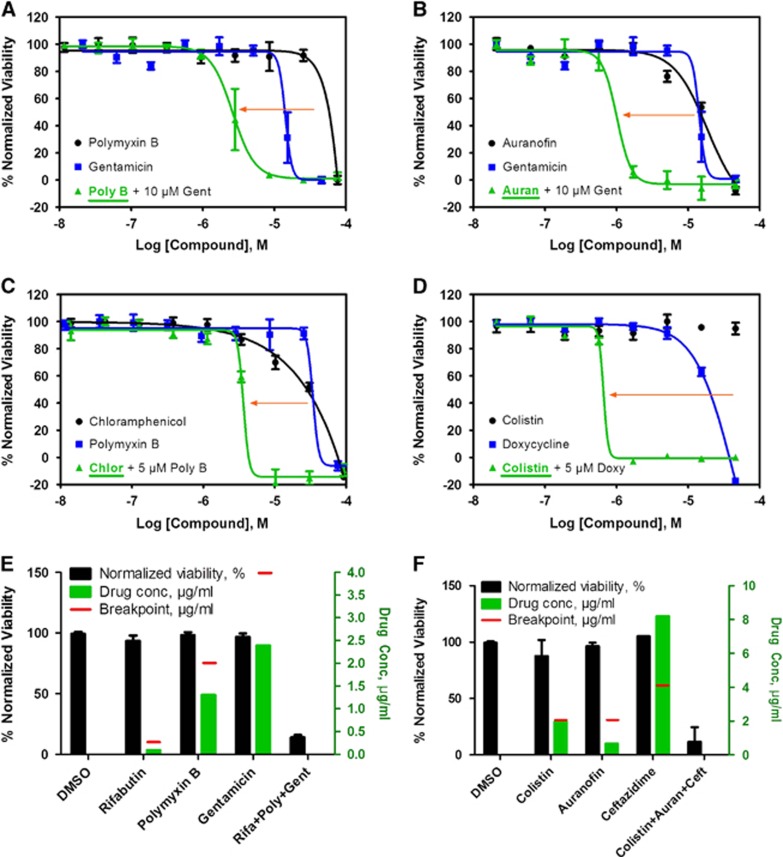
Active compounds re-sensitize *K. pneumoniae* KPNIH1760 to standard care antibiotics. In the drug combination conditions, compounds underlined are plotted in dose–response. Concentration–response curves were generated for the drug combinations indicated. KPNIH1760 was treated with gentamicin (Gent) (10 μM), polymyxin B (Poly B) (IC_20_) or doxycycline (Doxy) (IC_20_), combined with varying concentrations of polymixin B, auranofin (Auran), chloramphenicol (Chlor) or colistin for 24 h at 37 °C before detection of bacterial growth at OD_600_ (green line). (**A**, **B**) In combination with 10 μM gentamicin, IC_50_ of polymyxin B was improved from 44 to 2.62 μM (3.41 μg/mL) (IC_90_ of 5.74 μM (7.46 μg/mL)); IC_50_ of auranofin was improved from 20 to 1.01 μM (686 ng/mL) (IC_90_ of 1.67 μM (1.13 μg/mL)). (**C**) In combination with 5 μM polymyxin B, IC_50_ of chloramphenicol was lowered from 24 to 3.65 μM (1.18 μg/mL) (IC_90_: 4.47 μM (1.44 μg/mL)). (**D**) In combination with 5 μM doxycycline, IC_50_ of colistin was lowered from >46 μM to 658 nM (759 ng/mL) (IC_90_: 786 nM (909 ng/mL)). Three-drug synergistic targeted drug combinations (TDCs) against KPNIH1760 (**E**, **F**). Single drug, three-drug combination or dimethylsulphoxide (DMSO) (vehicle control) was plotted with drug concentration (green bars) and corresponding % normalized viability (black bars). Antimicrobial susceptibility breakpoint of the single drug was indicated (red line). (**E**) Synergistic bactericidal effect of the three-drug combination: rifabutin (Rifa)—0.09 μg/mL, polymyxin B (Poly)—1.3 μg/mL and gentamicin (Gent)—2.39 μg/mL against KPNIH1760. (**F**) Synergistic bactericidal effect of the three-drug combination: colistin—1.96 μg/mL, auranofin (Auran)—0.67 μg/mL and ceftazidime (Ceft)—8.20 μg/mL; *n*=4. Bar graph represent mean, and error bars represent the SEM.

For the 25 confirmed compounds, we found the maximum human plasma concentrations (*C*_max_) reported in the literature were often lower than their effective concentrations (IC_90_) against the four *K. pneumoniae* isolates (Table 2). The required concentrations of most of these 25 drugs for suppressing MDR *K. pneumoniae* growth *in vitro* are higher than the maximum peak concentration the drug can achieve in serum, which would prohibit clinical use. Of the 20 clinically tested antibiotics against MDR KPNIH1760 (Supplementary Table S1), only gentamicin showed activity at a high concentration (10.2 μg/mL) that can result in a severe side effect of bilateral vestibulopathy.^28^ To reduce the required drug concentration necessary to inhibit MDR *K. pneumoniae* growth, we designed a TDC strategy to test and identify drug combinations that show synergistic effects against MDRB. A two-drug combination was implemented, which paired a newly identified drug from our drug repurposing screen with one of the standard care antibiotics against *K. pneumoniae* that were ineffective against MDR *K. pneumoniae*. The effective drug combination would reduce the required concentration for each individual drug. The goal was to use a newly identified compound to re-sensitize KPNIH1760 to a standard care antibiotic. A total of 375 two-drug combinations were designed that tested 25 newly identified compounds and 15 clinically used antibiotics against *K. pneumoniae*. These 25 newly identified compounds consisted of 11 FDA-approved drugs (Figure 3) and 14 drugs used for investigational purposes, animal use and antiseptics (Supplementary Figure S2). We observed that the addition of 10 μM gentamicin (human plasma *C*_max_=20.9 μM) reduced the IC_50_ value of polymyxin B from 44 to 2.6 μM (17-fold; Figure 3A), and also reduced auranofin IC_50_ from 20 to 1.0 μM (20-fold) against MDR KPNIH1760 (Figure 3B). Though 5 μM polymyxin B alone showed <25% inhibition of KPNIH1760, it re-sensitized KPNIH1760 to chloramphenicol by sixfold (Figure 3C). Doxycycline also re-sensitized KPNIH1760 to colistin with the IC_50_ value of colistin alone at >45 μM, but only 0.66 μM when combined with doxycycline (Figure 3D). Standard microdilution broth assay confirmed the effectiveness of the two-drug combination gentamicin and polymixin B. The addition of gentamicin (1 μg/mL) reduced the MIC of polymixin B from 8 μg/mL down to 2 μg/mL for KPNIH1760. The above drug repurposing screen and drug combination antimicrobial susceptibility testing can be completed in one week with automation. However, the individual drug concentrations of some compounds in the two-drug combinations were still above the clinical antimicrobial susceptibility breakpoints or their maximum human plasma concentrations; although these two-drug combinations showed growth inhibition of KPNIH1760. For example, 5 μM auranofin was needed to re-sensitize KPNIH1760 to antibiotics, but that is still higher than the auranofin peak human plasma concentration (1 μM). The required gentamicin concentration in the two-drug combinations was 10 μM. Although its human plasma concentration can reach 20 μM, gentamicin has ototoxicity and nephrotoxicity after prolonged treatment at plasma concentration over 4 μM.^28^ We also found that dibromopropamidine, an antiseptic and disinfectant, re-sensitized KPNIH1760 to ciprofloxacin, trimethoprim, piperacillin/tazobactam, aztreonam and chloramphenicol (Supplementary Figure S2A, S2C, S2F, S2E and S2G). Meclocycline (a tetracycline antibiotic used topically for skin infections) and o-(chloromercuri)phenol (microbiocide) re-sensitized KPNIH1760 to colistin (Supplementary Figure S2B). Although these particular compounds cannot be administered orally or intravenously to humans, further study of these compounds may result in identification of mechanisms of drug resistance and new drug targets against MDR *K. pneumoniae*.

### Identification of three-drug combination against MDR *K. pneumoniae*

To identify clinically useful drug combinations with individual drug concentrations below reachable drugs' plasma concentrations, toxic dosages and clinical breakpoints, we applied the TDC to design three-drug combinations. On the basis of the two-drug synergistic combinations above, a third drug was added to form the three-drug combination. A total of 820 TDCs were screened against MDR KPNIH1760, and each drug had a distinct mechanism of action to maximize the synergistic effect and to avoid the additive toxic effect. The three-drug combinations were considered effective if >80% KPNIH1760 growth was suppressed. From this screen, a set of 17 three-drug combinations met the criteria (Figure 3 and Supplementary Table S3): all drugs are FDA-approved and nine of them are clinically used antibiotics. The 17 TDCs were divided into three major groups. The first group of eight combinations contained polymyxin B and rifabutin plus a third drug of gentamicin (Figure 3E), zidovudine, trimethoprim, aztreonam, ceftazidime, imipenem or ciprofloxacin. The second group of five combinations included auranofin and colistin plus a third drug of imipenem, gentamicin, rifabutin, ceftazidime or zidovudine (Figure 3F). The last group of four combinations included auranofin and polymyxin B plus a third drug of gentamicin, ceftazidime, rifabutin or imipenem. Although none of these drugs could achieve more than 30% efficacy individually at the given concentrations, 17 of the three-drug combinations exhibited 80% or more inhibition of KPNIH1760 growth. We also determined the MIC values of the auranofin, rifabutin and polymixin B three-drug combination against MDR KPNIH1760. The MIC data and IC_90_ data (Supplementary Table S3, combination KPTDC6) for this three-drug combination were the same.

### Identification of three-drug combinations with broad antibacterial spectrum against 10 common clinical MDRB strains

**Figure 4:**
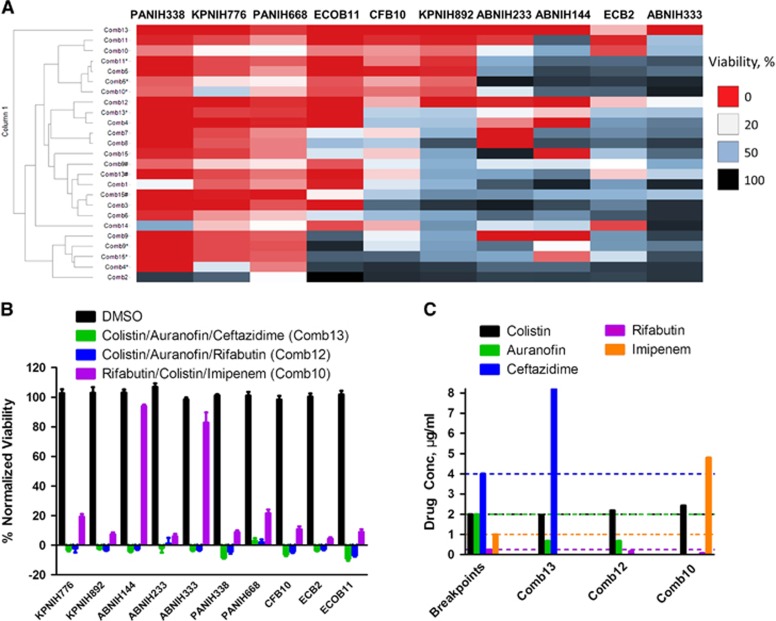
Broad-spectrum bactericidal effects of three-drug combinations. Fifteen three-drug targeted drug combinations (TDCs) were tested at their IC_90_ concentrations against 10 multidrug-resistant strains, including *K. pneumoniae* (KPNIH776 and KPNIH892), *A. baumannii* (ABNIH144, ABNIH233 and ABNIH333), *P. aeruginosa* (PANIH338 and PANIH668), *C. freundii* (CFB10), *E. cloacae* (ECB2) and *E. coli* (ECOB11). (**A**) Heatmap of bactericidal effects of 15 TDCs against 10 MDR bacteria; 0% viability (red), 20% viability (white), 50% viability (blue) and 100% viability (black). Comb1 (rifabutin—0.052 μM, polymyxin B—1 μM and zidovudine—1 μM); Comb2 (rifabutin—0.056 μM, polymyxin B—1 μM and trimethoprim—4 μM); Comb3 (rifabutin—0.056 μM, polymyxin B—1 μM and aztreonam—4 μM); Comb4 (rifabutin—0.06 μM, polymyxin B—1 μM and ceftazidime—15 μM); Comb4* (rifabutin—0.06 μM, polymyxin B—1 μM and ceftazidime—4 μM); Comb5 (rifabutin—0.09 μM, polymyxin B—1 μM and imipenem—16 μM); Comb5* (rifabutin—0.09 μM, polymyxin B—1 μM and imipenem—8 μM); Comb6 (rifabutin—0.052 μM, colistin—2.1 μM and zidovudine—1 μM); Comb7 (rifabutin—0.056 μM, colistin—2.1 μM and trimethoprim—4 μM); Comb8 (rifabutin—0.056 μM, colistin—2.1 μM and aztreonam—4 μM); Comb9 (rifabutin—0.06 μM, colistin—2.1 μM and ceftazidime—15 μM); Comb9* (rifabutin—0.06 μM, colistin—2.1 μM and ceftazidime—8 μM); Comb9# (rifabutin—0.06 μM, colistin—2.1 μM and ceftazidime—4 μM); Comb10 (rifabutin—0.09 μM, colistin—2.1 μM and imipenem—16 μM); Comb10* (rifabutin—0.09 μM, colistin—2.1 μM and imipenem—8 μM); Comb11 (colistin—1.2 μM, auranofin—1 μM and imipenem—16 μM); Comb11* (colistin—1.2 μM, auranofin—1 μM and imipenem—8 μM); Comb12 (colistin—1.9 μM, auranofin—1 μM and rifabutin—0.2 μM); Comb13 (colistin—1.7 μM, auranofin—1 μM and ceftazidime—15 μM); Comb13* (colistin—1.7 μM, auranofin—1 μM and ceftazidime—8 μM); Comb13# (colistin—1.7 μM, auranofin—1 μM and ceftazidime—4 μM); Comb14 (colistin—2.1 μM, auranofin—1 μM and zidovudine—1 μM); Comb15 (polymyxin B—1.8 μM, auranofin—1 μM and ceftazidime—15 μM); Comb15* (polymyxin B—1.8 μM, auranofin—1 μM and ceftazidime—8 μM); Comb15# (polymyxin B—1.8 μM, auranofin—1 μM and ceftazidime—4 μM); *n*=4; * and # represent the same drugs are in combination but with different concentrations. (**B**) Top three TDCs and dimethylsulphoxide (DMSO) control were plotted as % normalized viability of different MDR isolates: (i) colistin—1.96 μg/mL, auranofin—0.68 μg/mL and ceftazidime—8.20 μg/mL (green) (Comb13 in A; (ii) colistin—2.19 μg/mL, auranofin—0.68 μg/mL and rifabutin—0.17 μg/mL (blue) (Comb12 in A; (iii) rifabutin—0.08 μg/mL, colistin—2.43 μg/mL and imipenem—4.80 μg/mL (purple) (Comb10 in A; *n*=4. Bar graph represent mean, and error bars represent the SEM. (**C**) Clinical breakpoints^24, 29^ and drug concentrations in three-drug TDCs. Bars represent drug concentrations of colistin (black), auranofin (green), ceftazidime (blue), rifabutin (purple) and imipenem (orange), and corresponding colored dashed lines represent individual drug susceptibility breakpoints. Breakpoints were selected for auranofin^30^ and rifabutin^31^ based on the selected literature. Imipenem and ceftazadime breakpoints were based on CLSI guidelines for Enterobacteriaceae. Imipenem breakpoint is 2 μg/mL and ceftazadime breakpoint is 8 μg/mL for *Acinetobacter* spp. and *Pseudomonas* spp.^24^

To identify effective three-drug combinations against common clinical MDRB strains, we tested 15 combinations based on the above results against 10 clinically relevant Gram-negative MDR strains including *K. pneumoniae* (KPNIH776 and KPNIH892), *A. baumannii* (ABNIH144, ABNIH233 and ABNIH333), *P. aeruginosa* (PANIH338 and PANIH668), *C. freundii* (CFB10), *E. cloacae* (ECB2) and *E. coli* (ECOB11) (Table 1 and Figure 4A). At the individual drug concentrations below reported *C*_max_ values in human plasma and clinical breakpoints, none of the individual drugs at such concentrations suppressed these 10 MDR strains (Supplementary Table 4). We found that the combinations of Comb13 (colistin, auranofin and ceftazidime) and Comb12 (colistin, auranofin and rifabutin) (Figure 4B) suppressed >80% growth of all 10 MDR strains. The third effective combination Comb10 (rifabutin, colistin and imipenem) inhibited >75% of these strains except ABNIH144 and ABNIH333. All individual drug concentrations in these three-drug combinations were near or below clinical breakpoints with the exception of ceftazidime in the colistin/auranofin/ceftazadime combination and imipenem in the rifabutin/colistin/imipenem combination (Figure 4C). We employed the microdilution broth assay to confirm the three-drug combination auranofin, rifabutin and colistin. The MIC data showed a synergistic effect (FIC index=0.44) for the three-drug combination against *E. coli* ECOB11 compared with individual drugs (Table 3B). These results demonstrate that HIGA along with the TDC approach enables rapid identification of effective three-drug combinations with broad spectrum against multiple MDRB.

Finally, we tested whether the combination of three randomly chosen antibiotics with different mechanisms of action would have synergistic effects against an MDR strain without the use of HIGA and the TDC approach. Gentamicin, tetracycline and meropenem combination was selected as three-drug combination and the activity against intermediately resistant KPNIH535 was determined using the standard broth microdilution assay. We found that there was limited synergy with the two-drug combination of meropenem and gentamicin for KPNIH535 compared with individual drugs (Supplementary Table S5). The addition of the third drug tetracycline did not provide additional value. This suggests that HIGA and TDC approach can provide a benefit with the ability to screen large numbers of drug combinations to identify specific drugs and drug combinations that may have synergistic effects against MDR bacteria beyond what could be accomplished with traditional methods.

## Discussion

Multidrug resistance in bacteria has been rising markedly over the past decades.^2^ There are limited treatment options for patients with life-threatening infections as few new antibiotics have become available. We have developed HIGA to rapidly screen hundreds of approved drugs against clinical patient isolates to identify effective drugs and drug combinations. This method provides a new approach to overcome two current major limitations facing the infectious disease diagnostics field. First, HIGA quantitatively screens hundreds of approved drugs using patient isolates to rapidly identify effective therapeutics options against MDRB. Second, the TDC strategy assists designing drug combinations that maximize synergistic effects and reduce individual drug concentrations, which in turn reduces both the drug toxicity effects and the development of further drug resistance.

**Figure 5:**
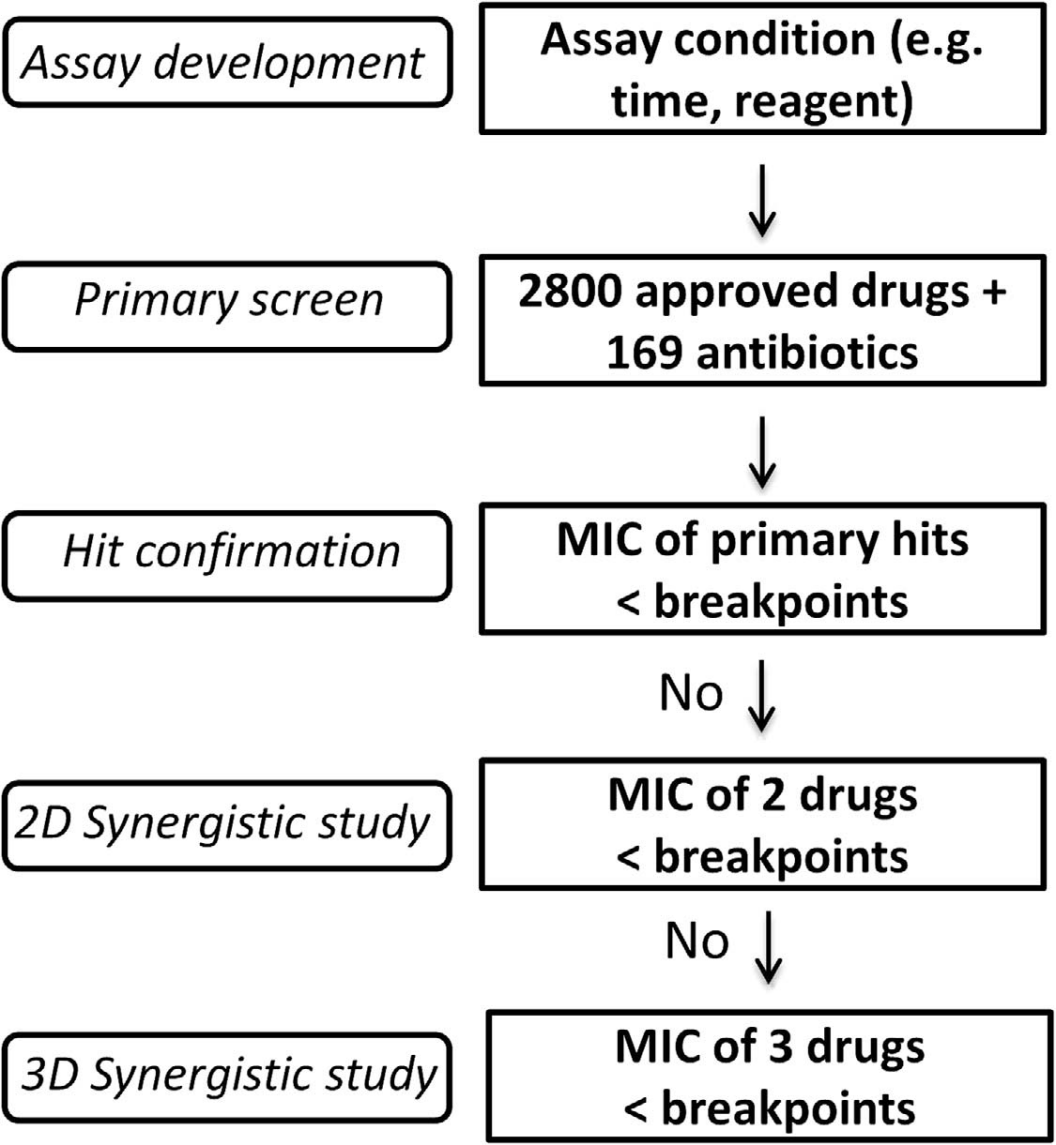
Flow chart of future rapid antimicrobial susceptibility tests for treatment of multidrug-resistant organisms. (i) The first step is assay development, including reaction time and assay reagents. (ii) The second step is large-scale screening of available antibiotics and other approved drugs. (iii) The third step is confirmation of hits: any hits with >90% suppression of MDRB growth and a drug concentration below its clinical microbial susceptibility breakpoint may be investigated. (iv) The fourth step (optional) is two-drug (2D) TDC using the above confirmed hits with MIC90 above the clinical breakpoints and commonly used antibiotics. A 2D synergistic combination will be further investigated if its combined bactericidal effect is more than 90% and the drug concentration of each individual drug is no more than its breakpoint. (v) The fifth step (optional) is three-drug (3D) TDC using above 2D combinations with the MIC_90_ above the clinical breakpoints and commonly used antibiotics. A 3D synergistic combination is proposed as a potentially effective therapeutic combination if its combined bactericidal effect is beyond 90% and the drug concentration of each individual drug is below its clinical breakpoint.

Inappropriate initial antibiotic therapies for infections with MDRB are associated with higher mortality rates.^6^ Many of these patients are in intensive care units (ICU), undergoing treatment with empirical antibiotics. In the future, clinicians could supplement the current standard antimicrobial treatment with additional drugs identified from the HIGA and TDC approach. Effective two- and three-antibiotic combinations identified with the above method can then be confirmed/validated using the traditional clinical laboratory tests (Figure 5), but that would only be a limited set of combinations, which would be manageable with traditional methods.

The main advantage of this drug repurposing screen approach is the identification and application of approved drugs for a new indication. In this case, antimicrobial compounds can move more quickly to clinical trials or treatments without a prolonged period of preclinical drug development.^32^ The primary screening and active compound confirmations can also be completed in 1–2 weeks. In this study, we identified 25 compounds with antibacterial activity against two MDR *K. pneumoniae* strains (KPNIH1760 and KPNIH1776). Among these hits, 10 out of 25 are antibiotics. The other 15 include the anti-HIV drug, zidovudine, which inhibits HIV DNA synthesis;^33^ an antimalarial drug, artesunate, which inhibits *Plasmodium falciparum* exported protein 1 (EXP1);^34^ two antirheumatics, auranofin and aurothioglucose, which inhibit IkappaB kinase and thioredoxin reductase;^35^ four antifungals, o-(chloromercuri)phenol, phenylmercuric acetate, zinc pyrithione, dipyrithione, which inhibit intracellular enzymes in fungus,^36^ inactivate copper influx or damage iron–sulfur proteins;^37^ an anticancer drug, bleomycin, which induce DNA strand breaks;^38^ an NADPH oxidase inhibitor, diphenyleneiodonium; and five antiseptics, thimerosal, phenylmercuric borate, dibromopropamidine, hexachlorophene and meclocycline, which inhibit intracellular enzymes,^36^ the electron transport pathway,^39^ or protein synthesis.^40^ Although these 15 hits will require additional investigation, validation, and extensive animal studies to examine the efficacy and toxicity before potential clinical use, these compounds may provide us with information for new targets and better insight into bacterial defense mechanisms for development of new antibiotics.

Combination antibiotic therapy has been used extensively to treat severe MDRB infections.^6^ Combination therapy can reduce mortality rates from 57.8% with monotherapy to 13.3% with combination therapy.^41^ The prudent use of antibiotic combinations will not only reduce development of resistance, but also may help reverse the prevalence of infections due to highly resistant organisms.^42^ There are two considerations for selecting clinically useful antibiotic combinations. First, the precise dosage of each individual antibiotic in combination is important. Finding the most effective, lowest dosage of antibiotics not only reduces cost and slows development of further resistance, but also decreases potential drug toxic effects, which is especially necessary for the vulnerable ICU patient population. Second, testing all the possible drug combinations is crucial but incredibly challenging due to the exponential number of possible combinations. It can be resolved by integration of this low-cost HIGA with the design of drug combinations based on individual drug concentrations below achievable human plasma concentrations, clinical susceptibility breakpoints, and different mechanisms of action (for example, TDC approach). Severe side-effects of 11 hits were summarized (Table 2) and clinicians will need to balance the benefits and potential toxicities of these drugs for treating infections. It is possible that a drug may not be used as monotherapy due to its toxicity at a high drug dosage or cannot reach effective plasma concentration but may be used in the drug combination described above.^43^ In this study, we identified and confirmed the three-drug combinations of colistin–auranofin–ceftazidime and colistin–auranofin–rifabutin with broad-spectrum activity at achievable individual drug plasma concentrations against ten MDRB strains including *K. pneumoniae*, *A. baumannii*, *P. aeruginosa*, *C. freundii*, *E. cloacae* and *E. coli*. A microbroth dilution assay confirmed the inhibition trend of colistin, auranofin and rifabutin against MDR *E. coli*.

Auranofin has potential to be used as treatment against infectious diseases.^44^ In a recent compound screening, auranofin exhibited efficacy against MDR *Streptococcus pneumoniae* and *Staphylococcus aureus* strains though it was ineffective against Gram-negative bacteria (MIC >16 μg/mL).^30^ However, we found that 1 μM (0.68 μg/mL) auranofin in combination with colistin and rifabutin inhibited 80% growth of ten MDR Gram-negative isolates. Another three-drug combination of rifabutin–colistin–imipenem also showed activities against 8 of these 10 MDRB. *Mycobacteria* and *Helicobacter* are often sensitive to rifamycins including rifampicin and rifabutin, but these drugs are ineffective against Enterobacteriaceae *in vitro*.^45^ In addition, a clinical trial showed no difference in 30- day mortality rates in patients treated with colistin or colistin plus rifampicin against extensively drug-resistant *A. baumannii*. However, the number of patients that eradicated *A. baumannii* was higher in the colistin plus rifampicin arm.^46^ Our screen suggests that the addition of a third drug to that particular two-drug combination may be beneficial for treatment.

There are well-documented limitations to *in vitro* combinational therapy testing. Different types of synergy tests give discrepant results, which make patient outcome analysis difficult to assess.^47^ In addition, there is simply a lack of blinded, controlled, randomized clinical trials to compare *in vitro* synergy data and *in vivo* patient outcomes. Although synergy testing is often used for MDRB infections in cystic fibrosis (CF) patients, there is only one reported controlled clinical trial, which concluded that CF patients did not benefit from synergy testing compared with conventional susceptibility testing.^48, 49^ Recently, a multi-center, multi-country randomized controlled study is underway to test the benefit of colistin in combination with meropenem compared with colistin alone based on promising *in vitro* synergy combination studies.^50^

Recently, personalized medicine or precision medicine has been extensively studied, especially in cancer therapies.^51^ We envision precision medicine applied to effective antimicrobial therapy for an individual patient. Interestingly, by comparing the activities of 11 hits (Table 2 and Supplementary Table S2) against the four *K. pneumoniae* isolates, three distinct subgroups were observed. Compounds in the first group, including bleomycin, gentamicin, sitafloxacin, garumonam, chloramphenicol and polymyxin B, were more effective against the susceptible isolates KPNIH301 and KPNIH478. Compounds in the second group, including flofenicol and rifabutin, had similar potency across four isolates. Compounds in the third group did not show a clear pattern: demeclocycline and doxycycline were more active against KPNIH301, less active against KPNIH1760 and KPNIH1776, inactive against drug sensitive KPNIH478; oxytetracycline were similarly active against KPNIH1760, KPNIH1776 and KPNIH301, while not active against KPNIH478. These results highlight the distinct drug activities against individual MDRB, indicating some compounds that are active to MDR isolates may not be equally active or are less active against the susceptible isolates of *K. pneumoniae*. HIGA enables rapid quantitative testing of hundreds of approved drugs against different MDRB to provide important information for personalized drug treatment.

Finally, many approved drugs may not be useful for the treatment of MDRB infections. For example, immunosuppressive agents may not be reasonable in combinations because patients require their host immune systems to combat the infections. A focused approved drug library with selected compounds should be a better approach for screening of clinical patient isolates to identify sensitive drugs and drug combinations. To our knowledge, <200 approved antibiotics are available.^11^ We are in the process of building an anti-MDRB library with selected approved drugs, including antibiotic, anti-viral, anti-fungal, anti-parasitic and other reported anti-infection drugs (for example, Auranofin).

In summary, we have developed an automated ultra-HIGA for antimicrobial susceptibility testing enabling rapid identification of effective therapeutics from approved drug collections for treatments of infections with MDRB. We identified 25 drugs that suppressed the growth of two drug-resistant *K. pneumoniae* strains. We also applied the TDC strategy to design two- and three-drug combinations against individual clinical MDR isolates and tested the drug combinations using HIGA. With this approach, three effective three-drug combinations were identified against ten clinically common MDRB strains. Therefore, HIGA potentially has broad applications to rapidly identify new therapeutics and effective drug-combinations against MDRB.

## Figures and Tables

**Table 1:** List of bacterial strains used in this study

**Strain**	**Description**	**Source**
KPNIH1760	*K. pneumoniae* patient isolate	NIH Clinical Center^21^
KPNIH1776	*K. pneumoniae* patient isolate	NIH Clinical Center
KPNIH301	*K. pneumoniae* patient isolate	NIH Clinical Center
KPNIH478	*K. pneumoniae* patient isolate	NIH Clinical Center
KPNIH535	*K. pneumoniae* patient isolate	NIH Clinical Center
KPNIH776	*K. pneumoniae* patient isolate	NIH Clinical Center
KPNIH892	*K. pneumoniae* patient isolate	NIH Clinical Center
ABNIH144	*A. baumannii* patient isolate	NIH Clinical Center
ABNIH233	*A. baumannii* patient isolate	NIH Clinical Center
ABNIH333	*A. baumannii* patient isolate	NIH Clinical Center
PANIH338	*P. aeruginosa* patient isolate	NIH Clinical Center
PANIH668	*P. aeruginosa* patient isolate	NIH Clinical Center
CFB10	*C. freundii* complex isolate	College of American Pathologists Breakpoint Implementation Toolkit, 2012
ECB2	*E. cloacae* isolate	College of American Pathologists Breakpoint Implementation Toolkit, 2012
ECOB11	*E. coli* isolate	College of American Pathologists Breakpoint Implementation Toolkit, 2012

**Table 2:** Activity of 11 FDA-approved drugs against four *Klebsiella pneumoniae* strains

**Drug**	**IC_50_ (μM) in four strains**				***C*_max_**	**Drug class**	**Mechanism of action**	**Severe side effect**
	**KPNIH1760**	**KPNIH1776**	**KPNIH301**	**KPNIH478**	**ng/mL (μM)**			
Bleomycin	2.3	2.4	0.3	0.5	600 (0.42)^a^	Anticancer	DNA metabolism	Pulmonary fibrosis, impaired lung function
Artesunate	10	3.7	NA	NA	3260 (8.50)	Antimalarial	Alkylation of heme	Rare
Gentamicin	14	6.3	1.8	1.07	10 000 (20.9)	Antibiotic	30S-subunit protein/16S rRNA	Nephrotoxicity, ototoxicity, low blood counts, allergic reactions, nerve damage
Demeclocycline	5.3	5.9	0.8	Inact.	1220 (2.62)	Antibiotic	Translation	Nephrogenic diabetes insipidus
Oxytetracycline	6.6	9.4	10	Inact.	2000 (4.34)	Antibiotic	Translation	Photosensitive allergic reactions, GI reactions
Zidovudine	6.5	3.7	0.1	0.32	300 (1.12)	Anti HIV	Transcription	Anemia, neutropenia, hepatotoxicity, cardiomyopathy, myopathy
Rifabutin	10	8.9	13	10	150 (0.18)	Antibiotic	DNA-dependent RNA polymerase	Skin allergic reactions, weakness, fever, easy bruising or bleeding
Chloramphenicol	15	15	1	1.26	10 000 (30.9)	Antibiotic	Protein synthesis	Aplastic anemia
Doxycycline	16	30	5	Inact.	5000 (11.2)	Antibiotic	Protein synthesis	Erythematous rash
Auranofin	20	19	7.9	7.94	680 (1.00)	Antirheumatics	Appab kinase/thioredoxin reductase	Allergic reaction, blood in urine and stools
Polymyxin B	44	32	1.6	2	13.9 (10.6)	Antibiotic	Altering membrane permeability	Allergic reactions

Abbreviations: <20% killing of *K. pneumoniae* at 46 μM, Inact.; inhibitory concentration of 50% response, IC_50_; not applicable (NA).

a The references for *C*_max_ are listed in Supplementary Table 6.

Note: KPNIH1760 and KPNIH1776 are resistant to 19/20 antibiotics tested, and KPNIH301 and KPNIH478 are sensitive to 16/18 antibiotics tested, respectively. Confirmed compounds were selected by a criteria of IC_50_ <50 μM and maximal inhibition >50%. For KPNIH1760, *n*=3, mean±SD.

**Table 3A:** Comparison of MIC values from the standard broth microdilution assay and IC_90_ values of active compounds against *K. pneumoniae* KPNIH1760

**Drug**	**MIC (μg/mL)**	**IC_90_-HIGA (μg/mL)**	**IC_50_-HIGA, μg/mL (μM)**
Bleomicin sulfate	0.7±0.3	5.0±2.0	3.3±0.6 (2.3±0.4)
Zidovudine	4±0.0	6.1±3.2	1.7±0.4 (6.5±1.4)
Rifabutin	32±0.0	32±21	8.5±6.9 (10±8.1)
Auranofin	64±0.0	27±2.8	13.6±0.6 (20±0.9)
Polymixin B	16±0.0	67±10	57±56 (44±43)
Gentamicin	3.3±1.2	10.2±0.6	8.2±1.2 (14.4±2.2)

Abbreviations: automated ultra-high-throughput bacterial growth assay, HIGA; minimum inhibitory concentration, MIC.

Note: IC_90_ values were calculated by the Prism software (*n*=3, mean±SD).

**Table 3B:** MIC values of three drugs against MDR *E. coli* ECOB11 from the standard broth microdilution assay

**ECOB11 MIC (μg/mL)**			**FIC index**	**Interpretation**
**Auranofin**	**Rifabutin**	**Colistin**		
64±0	NA	NA	NA	NA
NA	16±0	NA	NA	NA
NA	NA	2±0	NA	NA
1.0±0	2.8±1.1	0.5±0	0.4	Synergy

Abbreviations: fractional inhibitory concentration, FIC; not applicable, NA.

Note: *n*=5, mean±SD.
