# Supplementary material for: Rapid antimicrobial susceptibility test for identification of new therapeutics and drug combinations against multidrug-resistant bacteria
Source: Emerg Microbes Infect. 2016 Nov 9;5(11):e116–. doi: 10.1038/emi.2016.123 (PMC5148025; doi:10.1038/emi.2016.123)
Supplement: Supplementary Figure 1 [file emi2016123x1.pdf]

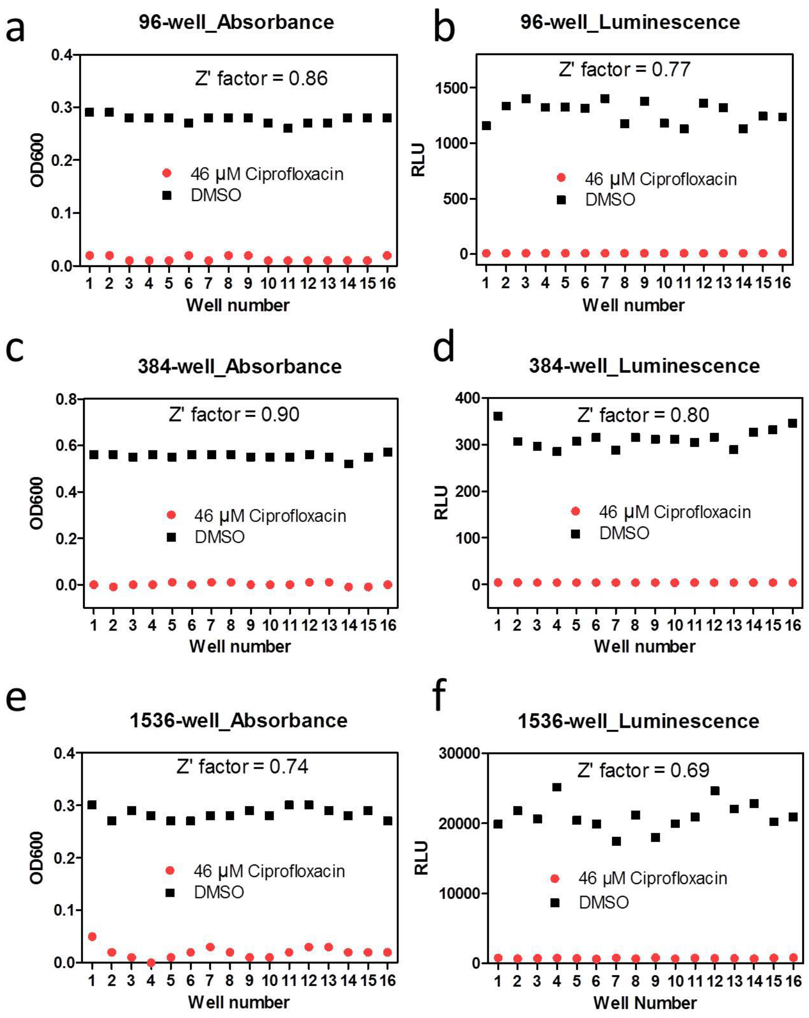

Supplementary Figure S1 Comparison of absorbance-based and luminescence-based assays with different plate types. KPNIH301 were incubated with either DMSO (black squares) or 46 µM ciprofloxacin (red circles) for 6 h at 37°C in 96 well plates (a and b), 384 well plates (c and d), or 1536 well plates (e and f). The absorbance (a, c, e) and luminescence (b, d, f) measurements were determined after addition of the luciferase detection reagent. Each condition was repeated 16 times. The statistical parameters Z′ are calculated based on equations defined in the literature[1].

[1]Zhang JH, Chung TDY, Oldenburg KR. A simple statistical parameter for use in evaluation and validation of high throughput screening assays. J Biomol Screen 1999 Apr; 4(2): 67-73.
