# Supplementary material for: Rapid antimicrobial susceptibility test for identification of new therapeutics and drug combinations against multidrug-resistant bacteria
Source: Emerg Microbes Infect. 2016 Nov 9;5(11):e116–. doi: 10.1038/emi.2016.123 (PMC5148025; doi:10.1038/emi.2016.123)
Supplement: Supplementary Figure 2 [file emi2016123x2.pdf]

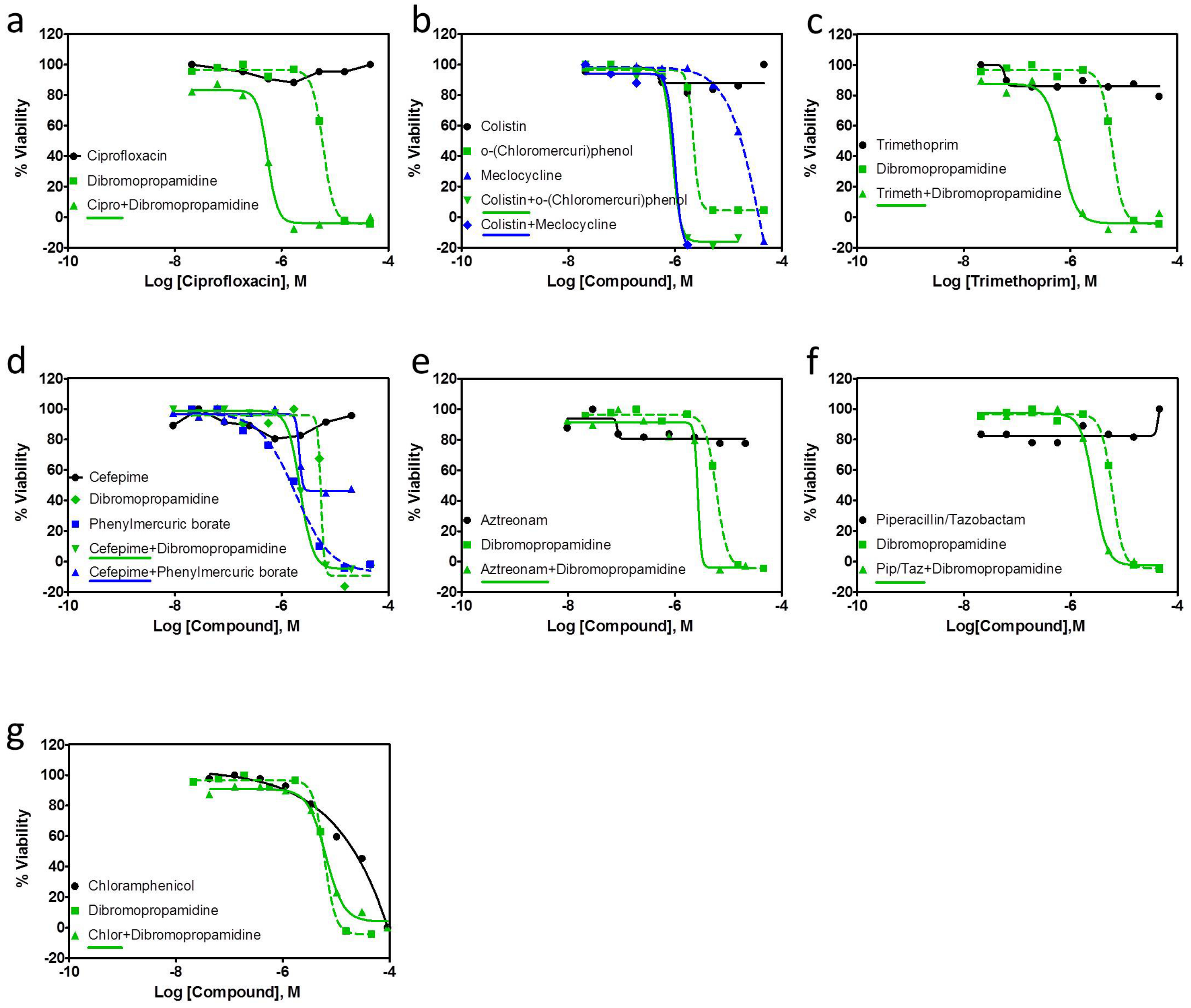

Supplementary Figure S2 Active compounds identified from repurposing screen re-sensitize MDR K. pneumoniae KPNIH1760 to clinically used antibiotics. In these drug combination conditions, compounds underlined are plotted as dose-response curves. Black or dashed lines represent concentration-response curves for single drugs: ciprofloxacin (a), colistin (b), trimethoprim (c), cefepime (d), aztreonam (e), piperacillin/tazobactam (f), or chloramphenicol (g) treatment against KPNIH1760. Newly identified active compounds were added to varying concentrations of these antibiotics (solid green or blue line) including: 5.1 μM dibromopropamidine (a, c, d, e, f, and g), 0.6 μM o-(chloromercuri)phenol (b), 2.3 μM meclocycline (b) 1.1 μM phenylmercuric borate (d). KPNIH1760 was treated with antibiotics and/or compounds for 24 h at 37°C before detection of bacterial growth at OD600. n=2. Abbreviations: Ciprofloxacin as Cipro, Trimethoprim as Trimeth, Chloramphenicol as Chlor, and Piperacillin/Tazobactam as Pip/Taz.
