# Supplementary material for: Rapid antimicrobial susceptibility test for identification of new therapeutics and drug combinations against multidrug-resistant bacteria
Source: Emerg Microbes Infect. 2016 Nov 9;5(11):e116–. doi: 10.1038/emi.2016.123 (PMC5148025; doi:10.1038/emi.2016.123)
Supplement: Supplementary Table 1 [file emi2016123x3.pdf]

Supplementary Table S1 IC_{50} and MIC data for standard of care antibiotics tested against *K. pneumoniae* strains

	KPNIH1760		KPNIH1776		KPNIH301		KPNIH478	
Drug name	IC_{50} (µM)	MIC (µg/ml)	IC_{50} (µM)	MIC (µg/ml)	IC_{50} (µM)	MIC (µg/ml)	IC_{50} (µM)	MIC (µg/ml)
Ampicillin	>45	>16	>45	>16	>45	>16	>45	>16
Cefazolin	>45	>16	>45	>16	7.4	≤8	2.1	≤2
Imipenem	>45	>8	>45	>8	33	≤4	30	≤1
Ceftazidime	>45	>2	>45	>2	6.3	≤8	2.2	≤0.5
Aztreonam	>45	>16	>45	>16	0.16	≤8	0.16	≤2
Cefepime	>45	>16	>45	>16	1.6	≤8	0.11	≤1
Trimethoprim	>45		>45		>45		>45	
Ciprofloxacin	>45	>2	>45	>2	3.9	≤1	0.11	≤0.5
Gentamicin	4.5	≤ 2	6.3	4.0	1.8	≤4	1.1	≤2
Meropenem	>45	>8	>45	>8	14	≤4	7.4	≤1
Amikacin	>45	32	>45	32	5.9	≤16	2.4	≤8
Cefuroxime	>45		>45			≤4	2	
Cefoxitin	>45	>16	>45	>16		≤8	4.9	≤4
Ceftriaxone	>45	>32	>45	>32		≤8	0.23	≤2
Levofloxacin	>45	>4	>45	>4		≤2	0.09	≤1
Nitrofurantoin	>45		>45			≤32	>45	32
Chloramphenicol	11	32	13		2.1		2.5	
Colistin	>45		>45					
Fosfomycin	>45		>45					
Piperacillin/ Tazobactam	>45	>64/4	>45	>64/4		≤16		4-4

Note: IC_{50} (µM) were calculated using customized software developed internally at National Center for Advancing Translations Sciences (NCATS) from 1536 well high throughput assay. MIC (µg/ml) was determined by automated susceptibility testing (BD Phoenix™).
