# Supplementary material for: Rapid antimicrobial susceptibility test for identification of new therapeutics and drug combinations against multidrug-resistant bacteria
Source: Emerg Microbes Infect. 2016 Nov 9;5(11):e116–. doi: 10.1038/emi.2016.123 (PMC5148025; doi:10.1038/emi.2016.123)
Supplement: Supplementary Table 2 [file emi2016123x4.pdf]

Supplmentary Table S2 Activity of 14 drugs approved for investigational purposes, animal use, and antiseptics against four *Klebsiella pneumoniae* strains

Drug Name	KPNIH1760 IC_{50} (µM)	KPNIH1776 IC_{50} (µM)	KPNIH301 IC_{50} (µM)	KPNIH478 IC_{50} (µM)	Cmax, (µM)	Drug Class	Mechanism of Action
Thimerosal	0.94	0.94	0.5	1.58	57.5 µg* Hg per dose	topical antiseptic, preservative in vaccines	releases ethyl-mercury inhibiting intracellular enzymes
o-(Chloromercuri)phenol	2.35	2.35	1.26	7.08		antifungal	
Phenylmercuric acetate	2.96	2.96	1	1.58		fungicide	releases ethyl-mercury inhibiting intracellular enzymes
Phenylmercuric borate	3.4	4.29	2.51	8.91		topical antiseptic	releases ethyl-mercury inhibiting intracellular enzymes
Florfenicol	4.48	2.83	3.16	3.98	16.5	antibiotic (animal)	inhibits protein synthesis
Sitafloxacin	7.43	6.31	0.25	0.05	11.3	antibiotic	inhibits DNA replication and transcription
Dibromopropamidine	9.35	9.35	12.59	7.94		antiseptic and disinfectant	
Zinc pyrithione	9.35	9.35	12.59	12.59		antifungal and antibacterial (topical)	Copper import and iron–sulfur proteins
Carumonam	9.35	9.35	0.08	0.06	145.8 Monkey	antibiotic	
Diphenyleneiodonium	9.35	7.43	2.51	2	0.528	NADPH oxidase inhibitors	NADPH oxidase inhibitors
Hexachlorophene	9.35	9.35	12.59	12.59	1.60	antiseptic	inhibits electron transport chain
Meclocycline	11.25	14.16	6.31	Inactive		antiseptic	inhibits protein synthesis
Dipyrithione	25.1	8.55	10	12.59		antifungal and antibacterial	Copper import and iron–sulfur proteins
Aurothioglucose	29.57	46.86	17.78	Inactive	11.5	rheumatoid arthritis	inhibits sulfhydryl systems

Note: KPNIH1760 and KPNIH1776 are resistant to 19/20 tested antibiotics, while KPNIH301 and KPNIH478 are sensitive to 16/18 antibiotics tested, respectively. Confirmed compounds were selected by a criteria of IC_{50} < 50 µM and maximal inhibition >50%. Abbreviations: IC_{50}: inhibitory concentration of 50% response; Inactive: <20% killing of *K. pneumoniae* at 46 µM. The references for Cmax are listed in SI Table 4.
