# Supplementary material for: Rapid antimicrobial susceptibility test for identification of new therapeutics and drug combinations against multidrug-resistant bacteria
Source: Emerg Microbes Infect. 2016 Nov 9;5(11):e116–. doi: 10.1038/emi.2016.123 (PMC5148025; doi:10.1038/emi.2016.123)
Supplement: Supplementary Table 3 [file emi2016123x5.pdf]

Supplementary Table S3 Summary of three-drug Targeted Drug Combination (TDC) against *K. pneumoniae* KPNIH1760

Combination #	Drug name-IC_{90} in Targeted Drug Combination (TDC)		
	Drug 1	Drug 2	Drug 3
KPTDC1	Rifabutin-0.02 µM	Polymyxin B-1 µM	Gentamicin-5 µM
Comb1	Rifabutin-0.04 µM	Polymyxin B-1 µM	Zidovudine-1 µM
Comb2	Rifabutin-0.06 µM	Polymyxin B-1 µM	Trimethoprim-4 µM
Comb3	Rifabutin-0.06 µM	Polymyxin B-1 µM	Aztreonam-18 µM
Comb4	Rifabutin-0.06 µM	Polymyxin B-1 µM	Ceftazidime-15 µM
Comb5	Rifabutin-0.09 µM	Polymyxin B-1 µM	Imipenem-16 µM
KPTDC2	Rifabutin-0.1 µM	Polymyxin B-1 µM	Ciprofloxacin-6 µM
KPTDC3	Rifabutin-0.1 µM	Polymyxin B-1 µM	Zidovudine-0.5 µM
Comb11	Colistin-1.2 µM	Auranofin-1 µM	Imipenem-16 µM
KPTDC4	Colistin-0.78 µM	Auranofin-1 µM	Gentamicin-5 µM
Comb12	Colistin-1.9 µM	Auranofin-1 µM	Rifabutin-0.2 µM
Comb13	Colistin-1.7 µM	Auranofin-1 µM	Ceftazidime-15 µM
Comb14	Colistin-2.1 µM	Auranofin-1 µM	Zidovudine-1 µM
KPTDC5	Polymyxin B-1.7 µM	Auranofin-1 µM	Gentamicin-5 µM
Comb15	Polymyxin B-1.7 µM	Auranofin-1 µM	Ceftazidime-15 µM
KPTDC6	Polymyxin B-2.1 µM	Auranofin-1 µM	Rifabutin-0.2 µM
KPTDC7	Polymyxin B-2.0 µM	Auranofin-1 µM	Imipenem-16 µM
