# Supplementary material for: Rapid antimicrobial susceptibility test for identification of new therapeutics and drug combinations against multidrug-resistant bacteria
Source: Emerg Microbes Infect. 2016 Nov 9;5(11):e116–. doi: 10.1038/emi.2016.123 (PMC5148025; doi:10.1038/emi.2016.123)
Supplement: Supplementary Table 5 [file emi2016123x7.pdf]

Supplementary Table S5 MIC data by standard broth microdilution assay of three randomly chosen drugs with different mechanisms of action: gentamicin, tetracycline, and meropenem against KPNIH535

KPNIH535 MIC (μg/mL)				
Gentamicin	Tetracycline	Meropenem	FIC Index	Interpretation
4 ± 0	NA	NA	NA	Sensitive
NA	8 ± 0	NA	NA	Intermediate
NA	NA	64 ± 0	NA	Resistant
2 ± 0	5.3 ± 2.3	NA	1.2	Indifference
1 ± 0	NA	16 ± 0	0.5	Synergy
NA	3.3 ± 1.2	21.3 ± 9.2	0.7	Indifference
1 ± 0	2.7 ± 1.2	16 ± 0	0.8	Indifference

FIC, fractional inhibitory concentration. NA, not applicable. n = 3, mean ± SD
